# Butyl 2-(3-benzoyl­thio­ureido)acetate

**DOI:** 10.1107/S1600536808033540

**Published:** 2008-10-22

**Authors:** Ibrahim N. Hassan, Bohari M. Yamin, Mohammad B. Kassim

**Affiliations:** a School of Chemical Sciences and Food Technology, Faculty of Science and Technology, Universiti Kebangsaan Malaysia, 43600 Bangi Selangor, Malaysia

## Abstract

In the title compound, C_14_H_18_N_2_O_3_S, the butyl acetate fragment and the benzoyl group adopt a *cis–trans* configuration, respectively, with respect to the thiono S atom across the C—N bonds. In the crystal packing, the mol­ecules are linked by inter­molecular N—H⋯O and C—H⋯O hydrogen bonds to form a one-dimensional chain along the *c* axis. The terminal butyl C atom is disordered with occupancies 0.82 (2)and 0.18 (2).

## Related literature

For information on bond lengths, see: Allen *et al.* (1987 ▶); For related structures, see: Hassan *et al.* (2008*a* ▶,*b* ▶); Yamin & Hassan (2004 ▶); Yamin & Yusof (2003 ▶).
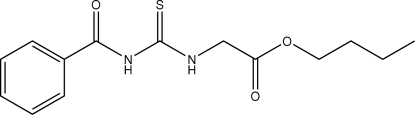


## Experimental

### 

#### Crystal data

C_14_H_18_N_2_O_3_S
*M* *_r_* = 294.36
Monoclinic, 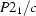

*a* = 14.051 (3) Å
*b* = 7.9482 (18) Å
*c* = 14.116 (3) Å
β = 102.753 (3)°
*V* = 1537.5 (6) Å^3^
*Z* = 4
Mo *K*α radiation
μ = 0.22 mm^−1^
*T* = 298 (2) K
0.46 × 0.28 × 0.25 mm

#### Data collection

Bruker SMART APEX CCD area-detector diffractometer
Absorption correction: multi-scan (*SADABS*; Sheldrick, 2004 ▶) *T*_min_ = 0.906, *T*_max_ = 0.947
7933 measured reflections
2853 independent reflections
2098 reflections with *I* > 2σ(*I*)
*R*_int_ = 0.022

#### Refinement

*R*[*F* ^2^ > 2σ(*F* ^2^)] = 0.049
*wR*(*F* ^2^) = 0.136
*S* = 1.03
2853 reflections
187 parameters
6 restraints
H-atom parameters constrained
Δρ_max_ = 0.24 e Å^−3^
Δρ_min_ = −0.27 e Å^−3^

### 

Data collection: *SMART* (Bruker, 2000 ▶); cell refinement: *SAINT* (Bruker, 2000 ▶); data reduction: *SAINT*; program(s) used to solve structure: *SHELXS97* (Sheldrick, 2008 ▶); program(s) used to refine structure: *SHELXL97* (Sheldrick, 2008 ▶); molecular graphics: *SHELXTL* (Sheldrick, 2008 ▶); software used to prepare material for publication: *SHELXTL*, *PARST* (Nardelli, 1995 ▶) and *PLATON* (Spek, 2003 ▶).

## Supplementary Material

Crystal structure: contains datablocks global, I. DOI: 10.1107/S1600536808033540/at2648sup1.cif

Structure factors: contains datablocks I. DOI: 10.1107/S1600536808033540/at2648Isup2.hkl

Additional supplementary materials:  crystallographic information; 3D view; checkCIF report

## Figures and Tables

**Table 1:** Hydrogen-bond geometry (Å, °)

*D*—H⋯*A*	*D*—H	H⋯*A*	*D*⋯*A*	*D*—H⋯*A*
N1—H1*A*⋯O2^i^	0.86	2.39	3.203 (2)	158
N2—H2*A*⋯O1	0.86	1.96	2.631 (2)	134
C2—H2⋯O1^i^	0.93	2.53	3.328 (3)	144

Symmetry code: (i) 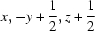.

**Fig. 1.**
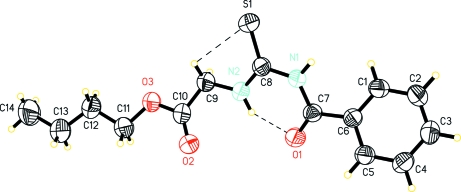
The molecular structure of (I) showing the main (80% occupancy) disorder component of the butyl terminal carbon atom, with displacement ellipsods are drawn at the 50% probability level.

**Fig. 2.**
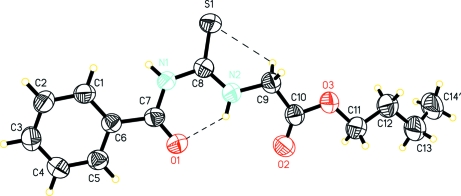
The molecular structure of (I) showing the minor (20% occupancy) disorder component of the butyl terminal carbon atom, with displacement ellipsods are drawn at the 50% probability level.

**Fig. 3.**
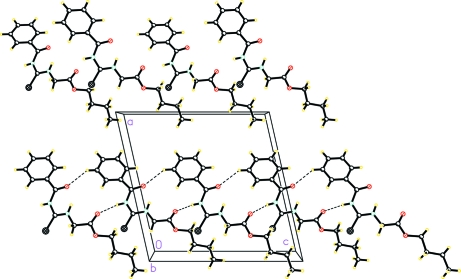
Crystal packing of (I) viewed down the a axis. Hydrogen bonds are drawn as dashed lines.

## supplementary crystallographic information

### Comment

The title compound (I) is a thiourea derivative of glycine analogous to ethyl-2-(3-benzoylthioureido)acetate (II) (Hassan *et al.*, 2008*a*) and propyl-2-(3-benzoylthioureido)acetate (III) (Hassan *et al.*, 2008*b*), with the shorter alkyl groups replaced by a butyl group. The molecule maintains the same *cis–trans* configuration with respect to the positions of the butyl acetate and benzoyl groups, relative to the S atom across the C—N bonds (Fig. 1 and Fig. 2), respectively. There is a disorder in the molecules involving the terminal butyl carbon atom. 'Soft' restrains, SIMU and EADP, were applied to the disorder components, C14 and C14', to resolve the overlapping components. In the final refinement the main disorder component, C14, resides in about 80% occupancy whereas the minor component, C14', occupies 20% at a time.

The compound was synthesized by a similar procedure to that of reported in (II). The bond lengths and angles in the molecules are in normal ranges (Allen *et al.*, 1987) and comparable to those in II and III. The phenyl ring, (C1–C6), and the central fragment, (C6/C7/C8/C9/N1/N2/S1), are essentially planar and the dihedral angle between them is 27.82 (9)°. In the butyl fragment, (C11/C12/C13/O3), the maximum deviation from the mean plane is 0.017 (3)Å for the atom C12. The dihedral angle between the phenyl ring and the butyl group is 14.5 (3)°. The intramolecular N2—H2···O1 and C9—H9B···S1 hydrogen bonds, (Table 1), force the molecule to adopt the present molecular conformation. The intermolecular N1—H1B···O2 and C2—H4A···O1 hydrogen bonds, (Table 2), link the molecules into a chain parallel to the *c* axis (Fig. 3).

### Experimental

Preparation of the compound was carried out according to a previously reported experimental procedures (Hassan *et al.*, 2008*a*). A yellowish crystal, suitable for X-ray crystallography, was obtained by a slow evaporation from CH_2_Cl_2_ solution at room temperature (yield 75%).

### Refinement

All H atoms were positioned geometrically and allowed to ride on their parent atoms, with, *U*_iso_ =1.2*U*_eq_ (N) for NH 0.86 Å, *U*_iso_=1.2*U*_eq_ (C) for aromatic 0.93 Å, *U*_iso_ = 1.2*U*_eq_ (C) for CH_2_ 0.97 Å and *U*_iso_ = 1.5*U*_eq_ (C) for CH_3_ 0.96 Å. The disordered component of the butyl group, C14 and C14', was resolved by applying SIMU and EADP constrains and refined anisotropically.

### Crystal data

**Table:**

C_14_H_18_N_2_O_3_S	*F*(000) = 624
*M**_r_* = 294.36	*D*_x_ = 1.272 Mg m^−^^3^
Monoclinic, *P*2_1_/*c*	Mo *K*α radiation, λ = 0.71073 Å
Hall symbol: -P 2ybc	Cell parameters from 2213 reflections
*a* = 14.051 (3) Å	θ = 3.0–25.5°
*b* = 7.9482 (18) Å	µ = 0.22 mm^−^^1^
*c* = 14.116 (3) Å	*T* = 298 K
β = 102.753 (3)°	Block, colourless
*V* = 1537.5 (6) Å^3^	0.46 × 0.28 × 0.25 mm
*Z* = 4	

### Data collection

**Table:**

Bruker SMART APEX CCD area-detector diffractometer	2853 independent reflections
Radiation source: fine-focus sealed tube	2098 reflections with *I* > 2σ(*I*)
graphite	*R*_int_ = 0.022
ω scans	θ_max_ = 25.5°, θ_min_ = 3.0°
Absorption correction: multi-scan (SADABS; Sheldrick, 2004)	*h* = −17→15
*T*_min_ = 0.906, *T*_max_ = 0.947	*k* = −9→9
7933 measured reflections	*l* = −17→16

### Refinement

**Table:**

Refinement on *F*^2^	Primary atom site location: structure-invariant direct methods
Least-squares matrix: full	Secondary atom site location: difference Fourier map
*R*[*F*^2^ > 2σ(*F*^2^)] = 0.049	Hydrogen site location: inferred from neighbouring sites
*wR*(*F*^2^) = 0.136	H-atom parameters constrained
*S* = 1.03	*w* = 1/[σ^2^(*F*_o_^2^) + (0.0696*P*)^2^ + 0.3732*P*] where *P* = (*F*_o_^2^ + 2*F*_c_^2^)/3
2853 reflections	(Δ/σ)_max_ < 0.001
187 parameters	Δρ_max_ = 0.24 e Å^−^^3^
6 restraints	Δρ_min_ = −0.26 e Å^−^^3^

### Special details

**Table:**

Geometry. All e.s.d.'s (except the e.s.d. in the dihedral angle between two l.s. planes) are estimated using the full covariance matrix. The cell e.s.d.'s are taken into account individually in the estimation of e.s.d.'s in distances, angles and torsion angles; correlations between e.s.d.'s in cell parameters are only used when they are defined by crystal symmetry. An approximate (isotropic) treatment of cell e.s.d.'s is used for estimating e.s.d.'s involving l.s. planes.
Refinement. Refinement of *F*^2^ against ALL reflections. The weighted *R*-factor *wR* and goodness of fit *S* are based on *F*^2^, conventional *R*-factors *R* are based on *F*, with *F* set to zero for negative *F*^2^. The threshold expression of *F*^2^ > σ(*F*^2^) is used only for calculating *R*-factors(gt) *etc*. and is not relevant to the choice of reflections for refinement. *R*-factors based on *F*^2^ are statistically about twice as large as those based on *F*, and *R*- factors based on ALL data will be even larger.

### Fractional atomic coordinates and isotropic or equivalent isotropic displacement parameters (Å^2^)

**Table:**

	*x*	*y*	*z*	*U*_iso_*/*U*_eq_	Occ. (<1)
C5	0.38240 (15)	0.4009 (3)	0.51713 (17)	0.0528 (6)	
H5	0.3785	0.4057	0.4506	0.063*	
C4	0.31404 (17)	0.4833 (3)	0.55659 (18)	0.0588 (6)	
H4	0.2645	0.5445	0.5167	0.071*	
C3	0.31902 (17)	0.4750 (3)	0.65504 (18)	0.0606 (6)	
H3	0.2730	0.5312	0.6816	0.073*	
C2	0.39148 (17)	0.3842 (3)	0.71373 (17)	0.0626 (7)	
H2	0.3936	0.3768	0.7799	0.075*	
C1	0.46139 (16)	0.3037 (3)	0.67530 (16)	0.0545 (6)	
H1	0.5114	0.2445	0.7158	0.065*	
C6	0.45697 (14)	0.3111 (3)	0.57640 (15)	0.0460 (5)	
C7	0.52769 (15)	0.2248 (3)	0.52830 (15)	0.0480 (5)	
C8	0.69838 (15)	0.1216 (3)	0.55880 (15)	0.0477 (5)	
C9	0.75837 (16)	−0.0258 (3)	0.43410 (16)	0.0563 (6)	
H9A	0.7547	−0.1438	0.4503	0.068*	
H9B	0.8220	0.0155	0.4671	0.068*	
C10	0.74863 (17)	−0.0094 (3)	0.32743 (17)	0.0526 (5)	
C11	0.82911 (19)	−0.0919 (4)	0.20303 (18)	0.0751 (8)	
H11A	0.8197	0.0214	0.1771	0.090*	
H11B	0.7797	−0.1641	0.1645	0.090*	
C12	0.92815 (19)	−0.1537 (4)	0.1994 (2)	0.0751 (8)	
H12A	0.9353	−0.2686	0.2231	0.090*	
H12B	0.9765	−0.0855	0.2424	0.090*	
C13	0.9475 (2)	−0.1483 (5)	0.0995 (2)	0.0897 (9)	
H13A	0.9217	−0.2484	0.0637	0.108*	
H13B	0.9161	−0.0507	0.0648	0.108*	
C14'	1.050 (2)	−0.139 (7)	0.108 (2)	0.111 (3)	0.18 (2)
H14E	1.0727	−0.0282	0.1288	0.166*	0.18 (2)
H14F	1.0652	−0.1633	0.0466	0.166*	0.18 (2)
H14D	1.0819	−0.2199	0.1554	0.166*	0.18 (2)
C14	1.0392 (5)	−0.2373 (17)	0.0885 (5)	0.111 (3)	0.82 (2)
H14B	1.0456	−0.2286	0.0223	0.166*	0.82 (2)
H14A	1.0356	−0.3537	0.1055	0.166*	0.82 (2)
H14C	1.0947	−0.1862	0.1306	0.166*	0.82 (2)
S1	0.80227 (5)	0.10476 (11)	0.64102 (5)	0.0744 (3)	
O1	0.50502 (11)	0.1805 (2)	0.44383 (11)	0.0647 (5)	
O2	0.68594 (12)	0.0656 (2)	0.27269 (13)	0.0709 (5)	
O3	0.82116 (12)	−0.0935 (2)	0.30338 (11)	0.0648 (5)	
N1	0.61986 (12)	0.2016 (2)	0.58446 (12)	0.0492 (5)	
H1A	0.6302	0.2413	0.6425	0.059*	
N2	0.68439 (13)	0.0652 (2)	0.46870 (13)	0.0517 (5)	
H2A	0.6292	0.0835	0.4295	0.062*	

### Atomic displacement parameters (Å^2^)

**Table:**

	*U*^11^	*U*^22^	*U*^33^	*U*^12^	*U*^13^	*U*^23^
C5	0.0497 (12)	0.0591 (14)	0.0492 (13)	−0.0022 (11)	0.0100 (10)	0.0037 (11)
C4	0.0504 (13)	0.0584 (15)	0.0658 (15)	0.0052 (11)	0.0090 (11)	0.0063 (12)
C3	0.0552 (14)	0.0631 (16)	0.0670 (16)	0.0002 (11)	0.0209 (12)	−0.0101 (12)
C2	0.0611 (15)	0.0801 (18)	0.0481 (13)	−0.0007 (13)	0.0154 (11)	−0.0050 (12)
C1	0.0499 (12)	0.0630 (15)	0.0481 (13)	0.0003 (11)	0.0057 (10)	0.0024 (11)
C6	0.0421 (11)	0.0488 (12)	0.0463 (12)	−0.0056 (9)	0.0077 (9)	−0.0015 (10)
C7	0.0460 (12)	0.0511 (13)	0.0459 (12)	−0.0034 (10)	0.0076 (9)	0.0015 (10)
C8	0.0470 (12)	0.0481 (13)	0.0495 (13)	−0.0026 (10)	0.0137 (10)	0.0069 (10)
C9	0.0539 (13)	0.0606 (15)	0.0566 (14)	0.0078 (11)	0.0171 (11)	0.0027 (11)
C10	0.0485 (12)	0.0540 (14)	0.0575 (14)	−0.0006 (11)	0.0162 (11)	−0.0009 (11)
C11	0.0707 (17)	0.101 (2)	0.0565 (15)	0.0157 (15)	0.0201 (13)	−0.0033 (14)
C12	0.0645 (16)	0.092 (2)	0.0718 (17)	0.0078 (14)	0.0222 (13)	−0.0051 (15)
C13	0.087 (2)	0.110 (2)	0.0789 (19)	0.0225 (18)	0.0322 (16)	0.0006 (17)
C14'	0.104 (3)	0.147 (7)	0.093 (3)	0.051 (4)	0.048 (3)	0.010 (4)
C14	0.104 (3)	0.147 (7)	0.093 (3)	0.051 (4)	0.048 (3)	0.010 (4)
S1	0.0534 (4)	0.1112 (6)	0.0549 (4)	0.0174 (4)	0.0040 (3)	−0.0017 (4)
O1	0.0538 (9)	0.0881 (12)	0.0482 (10)	0.0065 (9)	0.0030 (7)	−0.0130 (9)
O2	0.0631 (11)	0.0901 (13)	0.0620 (11)	0.0204 (10)	0.0188 (9)	0.0141 (9)
O3	0.0613 (10)	0.0803 (12)	0.0557 (10)	0.0175 (9)	0.0195 (8)	−0.0007 (8)
N1	0.0449 (10)	0.0609 (12)	0.0418 (9)	0.0013 (9)	0.0096 (7)	−0.0019 (8)
N2	0.0454 (10)	0.0605 (12)	0.0494 (11)	0.0029 (9)	0.0109 (8)	−0.0034 (9)

### Geometric parameters (Å, °)

**Table:**

C5—C4	1.377 (3)	C10—O2	1.195 (3)
C5—C6	1.386 (3)	C10—O3	1.324 (3)
C5—H5	0.9300	C11—O3	1.445 (3)
C4—C3	1.378 (3)	C11—C12	1.487 (4)
C4—H4	0.9300	C11—H11A	0.9700
C3—C2	1.368 (3)	C11—H11B	0.9700
C3—H3	0.9300	C12—C13	1.494 (4)
C2—C1	1.380 (3)	C12—H12A	0.9700
C2—H2	0.9300	C12—H12B	0.9700
C1—C6	1.385 (3)	C13—C14'	1.42 (3)
C1—H1	0.9300	C13—C14	1.507 (7)
C6—C7	1.489 (3)	C13—H13A	0.9700
C7—O1	1.216 (2)	C13—H13B	0.9700
C7—N1	1.373 (3)	C14'—H14E	0.9600
C8—N2	1.322 (3)	C14'—H14F	0.9600
C8—N1	1.389 (3)	C14'—H14D	0.9600
C8—S1	1.658 (2)	C14—H14B	0.9600
C9—N2	1.437 (3)	C14—H14A	0.9600
C9—C10	1.487 (3)	C14—H14C	0.9600
C9—H9A	0.9700	N1—H1A	0.8600
C9—H9B	0.9700	N2—H2A	0.8600
			
C4—C5—C6	120.2 (2)	O3—C11—H11B	110.1
C4—C5—H5	119.9	C12—C11—H11B	110.1
C6—C5—H5	119.9	H11A—C11—H11B	108.5
C5—C4—C3	120.0 (2)	C11—C12—C13	112.9 (2)
C5—C4—H4	120.0	C11—C12—H12A	109.0
C3—C4—H4	120.0	C13—C12—H12A	109.0
C2—C3—C4	120.1 (2)	C11—C12—H12B	109.0
C2—C3—H3	120.0	C13—C12—H12B	109.0
C4—C3—H3	120.0	H12A—C12—H12B	107.8
C3—C2—C1	120.4 (2)	C14'—C13—C12	108.2 (12)
C3—C2—H2	119.8	C14'—C13—C14	32.8 (19)
C1—C2—H2	119.8	C12—C13—C14	115.0 (3)
C2—C1—C6	119.9 (2)	C14'—C13—H13A	110.1
C2—C1—H1	120.0	C12—C13—H13A	110.1
C6—C1—H1	120.0	C14—C13—H13A	77.9
C1—C6—C5	119.4 (2)	C14'—C13—H13B	110.1
C1—C6—C7	123.66 (19)	C12—C13—H13B	110.1
C5—C6—C7	116.99 (19)	C14—C13—H13B	128.8
O1—C7—N1	122.4 (2)	H13A—C13—H13B	108.4
O1—C7—C6	121.58 (19)	C13—C14'—H14E	109.5
N1—C7—C6	115.97 (18)	C13—C14'—H14F	109.5
N2—C8—N1	116.64 (19)	C13—C14'—H14D	109.5
N2—C8—S1	124.56 (17)	C13—C14—H14B	109.5
N1—C8—S1	118.80 (16)	C13—C14—H14A	109.5
N2—C9—C10	112.83 (19)	H14B—C14—H14A	109.5
N2—C9—H9A	109.0	C13—C14—H14C	109.5
C10—C9—H9A	109.0	H14B—C14—H14C	109.5
N2—C9—H9B	109.0	H14A—C14—H14C	109.5
C10—C9—H9B	109.0	C10—O3—C11	118.49 (19)
H9A—C9—H9B	107.8	C7—N1—C8	127.81 (18)
O2—C10—O3	125.8 (2)	C7—N1—H1A	116.1
O2—C10—C9	126.0 (2)	C8—N1—H1A	116.1
O3—C10—C9	108.1 (2)	C8—N2—C9	122.18 (19)
O3—C11—C12	107.8 (2)	C8—N2—H2A	118.9
O3—C11—H11A	110.1	C9—N2—H2A	118.9
C12—C11—H11A	110.1		

### Hydrogen-bond geometry (Å, °)

**Table:**

*D*—H···*A*	*D*—H	H···*A*	*D*···*A*	*D*—H···*A*
N1—H1A···O2^i^	0.86	2.39	3.203 (2)	158
N2—H2A···O1	0.86	1.96	2.631 (2)	134
C2—H2···O1^i^	0.93	2.53	3.328 (3)	144
C9—H9B···S1	0.97	2.63	3.032 (2)	105

Symmetry codes: (i) *x*, −*y*+1/2, *z*+1/2.
